# Supplementary material for: Family history of Alzheimer’s disease alters cognition and is modified by medical and genetic factors
Source: eLife. 2019 Jun 18;8:e46179. doi: 10.7554/eLife.46179 (PMC6615857; doi:10.7554/eLife.46179)
Supplement: Source code 1. [file elife-46179-code1.pdf]

R Script

```
# Clears Environment "Careful"
rm(list = ls())

# Loads tidyverse package
library(tidyverse)
library(plyr)
# Loads Matt D.'s figure/graph theme
my_theme <- theme_bw() + theme(strip.text = element_text(face="bold", color="white",
size=rel(1.5)),
                    strip.background = element_rect(fill="black", color=NA, size=1),
                    plot.background = element_blank(),
                    panel.border = element_blank(),
                    panel.grid.major = element_blank(),
                    panel.grid.minor = element_blank(),
                    axis.title.x = element_text(face="bold", size=25),
                    axis.title.y = element_text(face="bold", size=25),
                    axis.text.x = element_text(size=15, angle=45, hjust=1),
                    axis.text.y = element_text(size=15),
                    legend.title = element_text(face="bold", size=12),
                    strip.text.x = element_text(size=20),
                    plot.title = element_text(lineheight=.8, face="bold",size=20),
                    axis.line = element_line(),
                    legend.key.size = unit(.75, "cm"),
                    legend.text = element_text(size=10))

# MindCrowd theme and color palette
mc_theme <- theme_bw() + theme(strip.text = element_text(face="bold", color="white",
size=rel(1.5)),
                    strip.background = element_rect(fill="black", color=NA, size=1),
                    plot.background = element_blank(),
                    panel.border = element_blank(),
                    panel.grid.major = element_blank(),
                    panel.grid.minor = element_blank(),
                    axis.title.x = element_text(face="bold", size=25),
                    axis.title.y = element_text(face="bold", size=25),
                    axis.text.x = element_text(size=15, angle=45, hjust=1),
                    axis.text.y = element_text(size=15),
                    legend.title = element_text(face="bold", size=12),
                    strip.text.x = element_text(size=20),
                    plot.title = element_text(lineheight=.8, face="bold",size=20),
                    axis.line = element_line(),
                    legend.key.size = unit(.75, "cm"),
                    legend.text = element_text(size=10))
```

```
mc_palette <- c("#FFB300", "#103FFF","#FF0074", "#97FF00", "#FFEF00")

# Reads in data from R's .rds file format from a specific after loading the readr package for MAC
df <-
readRDS("/Volumes/huentelman/mindcrowd2/data/mindcrowd_filtered_deident_20180815.rds")

# Removes outliers (those above perfect scores) in the total correct column in data frame df
df <- df[df$totalcorrect <=36, ]

prop.table(table(df$education)) * 100

ggplot(df, aes(age,totalcorrect)) +
  geom_point(alpha = 0.01, aes(color=df$age_halfdecade))+
  mc_theme

# Calculates proportions or percentages
prop.table(table (df$alzheimer))
prop.table(table (df$education))
prop.table(table (df$sex))

# Does Math
0.004341333+0.002786459+0.018119679+0.082623890

# All Variables to cut and paste
mylm <-lm(formula = totalcorrect ~ age + sex + marital_status + left_handed +
          education + number_of_daily_medications + diabetes + seizures +
          cancer + stroke + drug_abuse + loss_of_consciousness + dizziness +
          hypertension + heart_disease + smoking + alzheimer +race + hispanic_latino_or_spanish
        , data = df)
summary(mylm)


# All my MindCrowd linear models to write the results for our paper
# #1 Full Model with ALL demographic (RACE & ETHNICITY) vriables ALL AGES
df$race <- relevel(df$race, ref="White")
mylmFullAge <- lm(formula = totalcorrect ~ age + sex + marital_status + left_handed +
                  education + number_of_daily_medications + diabetes + seizures +
                  cancer + stroke + drug_abuse + loss_of_consciousness + dizziness +
                  hypertension + heart_disease + smoking + alzheimer + race +
hispanic_latino_or_spanish +
                  education*alzheimer + age*hypertension + sex*smoking + age*alzheimer +
age*diabetes +
                  alzheimer*diabetes + age*loss_of_consciousness +
                  sex*alzheimer*heart_disease
                , data = df)
```

```
summary(mylmFullAge)
plot(mylmFullAge)

# #2 Full Model with ALL demographic (RACE & ETHNICITY) variables < 65 years OLD
df$race <- relevel(df$race, ref="White")
mylmFull65 <- lm(formula = totalcorrect ~ age + sex + marital_status + left_handed +
                 education + number_of_daily_medications + diabetes + seizures +
                 cancer + stroke + drug_abuse + loss_of_consciousness + dizziness +
                 hypertension + heart_disease + smoking + alzheimer + race +
hispanic_latino_or_spanish +
                 education*alzheimer + age*hypertension + sex*smoking + age*alzheimer +
age*diabetes +
                 alzheimer*diabetes + age*loss_of_consciousness +
                 sex*alzheimer*heart_disease
               , data = df[df$age <= 65,])
summary(mylmFull65)
plot(mylmFull65)

# #3 Full Model with ALL demographic (RACE & ETHNICITY) variables FH AGE <65 APOE
FH
df$APOE <- as.factor((df$APOE))
df$APOE <- relevel(df$APOE, ref="2_3")
mylmAPOE <- lm(formula = totalcorrect ~ age + sex + marital_status + left_handed +
               education + number_of_daily_medications + diabetes + seizures +
               cancer + stroke + drug_abuse + loss_of_consciousness + dizziness +
               hypertension + heart_disease + smoking + alzheimer + hispanic_latino_or_spanish +
               education*alzheimer + age*hypertension + sex*smoking + age*alzheimer +
age*diabetes +
               alzheimer*diabetes + age*loss_of_consciousness +
               sex*alzheimer*heart_disease + APOE
             , data = df[df$age <=65 & df$alzheimer==TRUE,])
summary(mylmAPOE)
table(df$race, df$APOE)
table(df$hispanic_latino_or_spanish, df$APOE)

# #4 Full Model with ALL demographic (RACE & ETHNICITY) variables <65 FH
APOE_alleles
mylmAPOE_al <- lm(formula = totalcorrect ~ age + sex + marital_status + left_handed +
                 education + number_of_daily_medications + diabetes + seizures +
                 cancer + stroke + drug_abuse + loss_of_consciousness + dizziness +
                 hypertension + heart_disease + smoking + alzheimer + hispanic_latino_or_spanish +
                 education*alzheimer + age*hypertension + sex*smoking + age*alzheimer +
age*diabetes +
                 alzheimer*diabetes + age*loss_of_consciousness +
                 sex*alzheimer*heart_disease + APOE_alleles
               , data = df[df$age <=65 & df$alzheimer==TRUE,])
```

```
summary(mylmAPOE_al)

# #5 Full Model with ALL demographic (RACE & ETHNICITY) variables <65 FH
APOE_alleles Interaction
mylmAPOE_alINT <- lm(formula = totalcorrect ~ age + sex + marital_status + left_handed +
                  education + number_of_daily_medications + diabetes + seizures +
                  cancer + stroke + drug_abuse + loss_of_consciousness + dizziness +
                  hypertension + heart_disease + smoking + alzheimer + hispanic_latino_or_spanish
+ APOE_alleles +
                  education*alzheimer + age*hypertension + sex*smoking + age*alzheimer +
age*diabetes +
                  alzheimer*diabetes + age*loss_of_consciousness +
                  sex*alzheimer*heart_disease*APOE_alleles
                 , data = df[df$age <=65 & df$alzheimer==TRUE,])
summary(mylmAPOE_alINT)

# #6 Full Model with ALL demographic (RACE & ETHNICITY) variables < 65 years OLD
SUBSET for EMMEANS
df2 <- df[df$age <= 65,]
df2$race <- relevel(df2$race, ref="White")
mylmFull652 <- lm(formula = totalcorrect ~ age + sex + marital_status + left_handed +
               education + number_of_daily_medications + diabetes + seizures +
               cancer + stroke + drug_abuse + loss_of_consciousness + dizziness +
               hypertension + heart_disease + smoking + alzheimer + race +
hispanic_latino_or_spanish +
               education*alzheimer + age*hypertension + sex*smoking + age*alzheimer +
age*diabetes +
               alzheimer*diabetes + age*loss_of_consciousness +
               sex*alzheimer*heart_disease
              , data = df2)
summary(mylmFull652)

library(emmeans)

# Full Model EMM for 18-65 FH x DIABETES
emmmylmFull65FHDia <- emmeans(mylmFull652, ~ alzheimer*diabetes)
summary(emmmylmFull65FHDia)
plot(emmmylmFull65FHDia, comparisons = TRUE)
pairs(emmmylmFull65FHDia)

# Full Model EMM for 18-65 SEX x HD X FH
emmmylmFull65SexFHHD <- emmeans(mylmFull652, ~ sex*heart_disease*alzheimer)
summary(emmmylmFull65SexFHHD)
plot(emmmylmFull65SexFHHD, comparisons = TRUE)
pairs(emmmylmFull65SexFHHD)
```

```
# Full Model EMM for 18-85
emmmylmFull65SexFHHD2 <- emmeans(mylmFullAge, ~ sex*heart_disease*alzheimer)
summary(emmmylmFull65SexFHHD2)
plot(emmmylmFull65SexFHHD2, comparisons = TRUE)
pairs(emmmylmFull65SexFHHD2)

# ALTERED MODEL TO TEST: ALL demographic (RACE & ETHNICITY) Vriables Two-
Way Interactions
mylmAPOE2Way <- lm(formula = totalcorrect ~ age + sex + marital_status + left_handed +
              education + number_of_daily_medications + diabetes + seizures +
              cancer + stroke + drug_abuse + loss_of_consciousness + dizziness +
              hypertension + heart_disease + smoking + alzheimer + hispanic_latino_or_spanish +
              education*alzheimer + age*hypertension + sex*smoking + age*alzheimer +
age*diabetes +
              alzheimer*diabetes + age*loss_of_consciousness +
              sex*alzheimer + sex*heart_disease + alzheimer*heart_disease
             , data = df[df$age <=65,])
summary(mylmAPOE2Way)

# #2 Full Model with ALL demographic (RACE & ETHNICITY) variables 65-85 years OLD
df3 <- df[df$age >=65,]
df3$race <- relevel(df3$race, ref="White")
mylmFull6585 <- lm(formula = totalcorrect ~ age + sex + marital_status + left_handed +
               education + number_of_daily_medications + diabetes + seizures +
               cancer + stroke + drug_abuse + loss_of_consciousness + dizziness +
               hypertension + heart_disease + smoking + alzheimer + race +
hispanic_latino_or_spanish +
               education*alzheimer + age*hypertension + sex*smoking + age*alzheimer +
age*diabetes +
               alzheimer*diabetes + age*loss_of_consciousness +
               sex*alzheimer*heart_disease
              , data = df3)
summary(mylmFull6585)
plot(mylmFull6585)

# Full Model EMM for 18-65 SEX x HD X FH
emmmylmFull6585SexFHHD <- emmeans(mylmFull16585, ~ sex*heart_disease*alzheimer)
summary(emmmylmFull6585SexFHHD)
plot(emmmylmFull6585SexFHHD, comparisons = TRUE)
pairs(emmmylmFull6585SexFHHD)

# ALTERED MODEL TO TEST: ALL demographic (RACE & ETHNICITY) Variables Two-
Way Interactions
mylmAPOE2Way <- lm(formula = totalcorrect ~ age + sex + marital_status + left_handed +
                education + number_of_daily_medications + diabetes + seizures +
                cancer + stroke + drug_abuse + loss_of_consciousness + dizziness +
```

```
                 hypertension + heart_disease + smoking + alzheimer + hispanic_latino_or_spanish
+
                 education*alzheimer + age*hypertension + sex*smoking + age*alzheimer +
age*diabetes +
                 alzheimer*diabetes + age*loss_of_consciousness +
                 sex*alzheimer + sex*heart_disease + alzheimer*heart_disease
               , data = df2)
summary(mylmAPOE2Way)

library(tidyverse)
library(MatchIt)
library(Zelig)
set.seed(15)

load('../../mc.mn.sub.RData')

#
mc.df_20 = subset(mc.df,age < 25)
mc.df_30 = subset(mc.df,age >= 30 & age < 35)
mc.df_40 = subset(mc.df,age >= 40 & age < 45)
mc.df_50 = subset(mc.df,age >= 50 & age < 55)
mc.df_60 = subset(mc.df,age >= 60 & age < 65)
mc.df_70 = subset(mc.df,age >= 70 & age < 75)
mc.df_80 = subset(mc.df,age >= 80)

mc.df_25 = subset(mc.df,age >= 25 & age < 30)
mc.df_35 = subset(mc.df,age >= 35 & age < 40)
mc.df_45 = subset(mc.df,age >= 45 & age < 50)
mc.df_55 = subset(mc.df,age >= 55 & age < 60)
mc.df_65 = subset(mc.df,age >= 65 & age < 70)
mc.df_75 = subset(mc.df,age >= 75 & age < 80)
#
#
final.df = data.frame(variable=NA,ATT=NA,sample_size=NA,SD=NA,SE=NA)
n=1

##_20
pdf('sexFemale_20_propensity.pdf')
m.out = matchit(sexFemale ~
 age+marital_statussingle+marital_statusunreported+marital_statuswidowed+number_of_daily_
 medications+education_bin..20+education_bin13_19+alzheimerTRUE+hispanic_latino_or_spa
 nishTrue+left_handedTrue+drug_abuseTRUE+cancerTRUE+diabetesTRUE+dizzinessTRUE+h
 eart_diseaseTRUE+hypertensionTRUE+loss_of_consciousnessTRUE+seizuresTRUE+smoking
 TRUE+strokeTRUE,data=mc.df_20, method = "nearest",discard="both")
z.out.sexFemale = zelig(totalcorrect ~
 age+marital_statussingle+number_of_daily_medications+education_bin..20+education_bin13_
```

```
 19+sexFemale+alzheimerTRUE+hispanic_latino_or_spanishTrue+left_handedTrue+drug_abus
 eTRUE+cancerTRUE+diabetesTRUE+dizzinessTRUE+heart_diseaseTRUE+hypertensionTRU
 E+loss_of_consciousnessTRUE+seizuresTRUE+smokingTRUE,data = m.out,model =
 'ls',bootstrap=1000)
sink('sexFemale_20_summary.txt', append=FALSE, split=FALSE)
summary(z.out.sexFemale)
sink()
z.att.sexFemale_20 = z.out.sexFemale %>% ATT(treatment = 'sexFemale', treat = 1) %>%
 get_qi(qi = 'ATT', xvalue = 'TE')
final.df[n,"variable"] = "sexFemale_20"
final.df[n,"ATT"] = mean(z.att.sexFemale_20)
plot(m.out,type='hist')
hist(z.att.sexFemale_20,main = 'sexFemale_20',xlab='ATT')
dev.off()
#
#
m.data=match.data(m.out)
ss = nrow(match.data(m.data))
sd_ = sd(z.att.sexFemale_20)
se = sd_/sqrt(ss)
final.df[n,"sample_size"] = ss
final.df[n,"SD"] = sd_
final.df[n,"SE"] = se
n=n+1
#
View(final.df)
rm(z.out.sexFemale)
#
sex_20.df = data.frame(grp = '20',att = as.vector(z.att.sexFemale_20))
write.table(sex_20.df,'sex_20.long.csv',sep=',',col.names=T,row.names = F)
#
# ##_30
# pdf('sexFemale_30_propensity.pdf')
# m.out = matchit(sexFemale ~
 age+marital_statussingle+marital_statusunreported+marital_statuswidowed+number_of_daily_
 medications+education_bin..20+education_bin13_19+alzheimerTRUE+hispanic_latino_or_spa
 nishTrue+left_handedTrue+drug_abuseTRUE+cancerTRUE+diabetesTRUE+dizzinessTRUE+h
 eart_diseaseTRUE+hypertensionTRUE+loss_of_consciousnessTRUE+seizuresTRUE+smoking
 TRUE+strokeTRUE,data=mc.df_30, method = "nearest",discard="both")
# z.out.sexFemale = zelig(totalcorrect ~
 age+marital_statussingle+number_of_daily_medications+education_bin..20+education_bin13_
 19+sexFemale+alzheimerTRUE+hispanic_latino_or_spanishTrue+left_handedTrue+drug_abus
 eTRUE+cancerTRUE+diabetesTRUE+dizzinessTRUE+heart_diseaseTRUE+hypertensionTRU
 E+loss_of_consciousnessTRUE+seizuresTRUE+smokingTRUE,data = m.out,model =
 'ls',bootstrap=1000)
# sink('sexFemale_30_summary.txt', append=FALSE, split=FALSE)
```

```
# summary(z.out.sexFemale)
# sink()
# z.att.sexFemale_30 = z.out.sexFemale %>% ATT(treatment = 'sexFemale', treat = 1) %>%
 get_qi(qi = 'ATT', xvalue = 'TE')
# final.df[n,"variable"] = "sexFemale_30"
# final.df[n,"ATT"] = mean(z.att.sexFemale_30)
# plot(m.out,type='hist')
# hist(z.att.sexFemale_30,main = 'sexFemale_30',xlab='ATT')
# dev.off()
# #
# #
# m.data=match.data(m.out)
# ss = nrow(match.data(m.data))
# sd_ = sd(z.att.sexFemale_30)
# se = sd_/sqrt(ss)
# final.df[n,"sample_size"] = ss
# final.df[n,"SD"] = sd_
# final.df[n,"SE"] = se
# n=n+1
# #
# View(final.df)
# rm(z.out.sexFemale)
# #
# sex_30.df = data.frame(grp = '30',att = as.vector(z.att.sexFemale_30))
# write.table(sex_30.df,'sex_30.long.csv',sep=',',col.names=T,row.names = F)
#
# #
# ##_40
# pdf('sexFemale_40_propensity.pdf')
# m.out = matchit(sexFemale ~
 age+marital_statussingle+marital_statusunreported+marital_statuswidowed+number_of_daily_
 medications+education_bin..20+education_bin13_19+alzheimerTRUE+hispanic_latino_or_spa
 nishTrue+left_handedTrue+drug_abuseTRUE+cancerTRUE+diabetesTRUE+dizzinessTRUE+h
 eart_diseaseTRUE+hypertensionTRUE+loss_of_consciousnessTRUE+seizuresTRUE+smoking
 TRUE+strokeTRUE,data=mc.df_40, method = "nearest",discard="both")
# z.out.sexFemale = zelig(totalcorrect ~
 age+marital_statussingle+number_of_daily_medications+education_bin..20+education_bin13_
 19+sexFemale+alzheimerTRUE+hispanic_latino_or_spanishTrue+left_handedTrue+drug_abus
 eTRUE+cancerTRUE+diabetesTRUE+dizzinessTRUE+heart_diseaseTRUE+hypertensionTRU
 E+loss_of_consciousnessTRUE+seizuresTRUE+smokingTRUE,data = m.out,model =
 'ls',bootstrap=1000)
# sink('sexFemale_40_summary.txt', append=FALSE, split=FALSE)
# summary(z.out.sexFemale)
# sink()
# z.att.sexFemale_40 = z.out.sexFemale %>% ATT(treatment = 'sexFemale', treat = 1) %>%
 get_qi(qi = 'ATT', xvalue = 'TE')
```

```
# final.df[n,"variable"] = "sexFemale_40"
# final.df[n,"ATT"] = mean(z.att.sexFemale_40)
# plot(m.out,type='hist')
# hist(z.att.sexFemale_40,main = 'sexFemale_40',xlab='ATT')
# dev.off()
# #
# #
# m.data=match.data(m.out)
# ss = nrow(match.data(m.data))
# sd_ = sd(z.att.sexFemale_40)
# se = sd_/sqrt(ss)
# final.df[n,"sample_size"] = ss
# final.df[n,"SD"] = sd_
# final.df[n,"SE"] = se
# n=n+1
# #
# View(final.df)
# rm(z.out.sexFemale)
# #
# sex_40.df = data.frame(grp = '40',att = as.vector(z.att.sexFemale_40))
# write.table(sex_40.df,'sex_40.long.csv',sep=',',col.names=T,row.names = F)
# #
# #
# ##_50
# pdf('sexFemale_50_propensity.pdf')
# m.out = matchit(sexFemale ~
 age+marital_statussingle+marital_statusunreported+marital_statuswidowed+number_of_daily_
 medications+education_bin..20+education_bin13_19+alzheimerTRUE+hispanic_latino_or_spa
 nishTrue+left_handedTrue+drug_abuseTRUE+cancerTRUE+diabetesTRUE+dizzinessTRUE+h
 eart_diseaseTRUE+hypertensionTRUE+loss_of_consciousnessTRUE+seizuresTRUE+smoking
 TRUE+strokeTRUE,data=mc.df_50, method = "nearest",discard="both")
# z.out.sexFemale = zelig(totalcorrect ~
 age+marital_statussingle+marital_statuswidowed+number_of_daily_medications+education_bi
 n..20+education_bin13_19+sexFemale+alzheimerTRUE+hispanic_latino_or_spanishTrue+left_
 handedTrue+drug_abuseTRUE+cancerTRUE+diabetesTRUE+dizzinessTRUE+heart_diseaseT
 RUE+hypertensionTRUE+loss_of_consciousnessTRUE+seizuresTRUE+smokingTRUE+stroke
 TRUE,data = m.out,model = 'ls',bootstrap=1000)
# sink('sexFemale_50_summary.txt', append=FALSE, split=FALSE)
# summary(z.out.sexFemale)
# sink()
# z.att.sexFemale_50 = z.out.sexFemale %>% ATT(treatment = 'sexFemale', treat = 1) %>%
 get_qi(qi = 'ATT', xvalue = 'TE')
# final.df[n,"variable"] = "sexFemale_50"
# final.df[n,"ATT"] = mean(z.att.sexFemale_50)
# plot(m.out,type='hist')
# hist(z.att.sexFemale_50,main = 'sexFemale_50',xlab='ATT')
```

```
# dev.off()
# #
# #
# m.data=match.data(m.out)
# ss = nrow(match.data(m.data))
# sd_ = sd(z.att.sexFemale_50)
# se = sd_/sqrt(ss)
# final.df[n,"sample_size"] = ss
# final.df[n,"SD"] = sd_
# final.df[n,"SE"] = se
# n=n+1
# #
# View(final.df)
# rm(z.out.sexFemale)
# #
# sex_50.df = data.frame(grp = '50',att = as.vector(z.att.sexFemale_50))
# write.table(sex_50.df,'sex_50.long.csv',sep=',',col.names=T,row.names = F)
# #
# #
# ##_60
# pdf('sexFemale_60_propensity.pdf')
# m.out = matchit(sexFemale ~
 age+marital_statussingle+marital_statusunreported+marital_statuswidowed+number_of_daily_
 medications+education_bin..20+education_bin13_19+alzheimerTRUE+hispanic_latino_or_spa
 nishTrue+left_handedTrue+drug_abuseTRUE+cancerTRUE+diabetesTRUE+dizzinessTRUE+h
 eart_diseaseTRUE+hypertensionTRUE+loss_of_consciousnessTRUE+seizuresTRUE+smoking
 TRUE+strokeTRUE,data=mc.df_60, method = "nearest",discard="both")
# z.out.sexFemale = zelig(totalcorrect ~
 age+marital_statussingle+marital_statuswidowed+number_of_daily_medications+education_bi
 n..20+education_bin13_19+sexFemale+alzheimerTRUE+hispanic_latino_or_spanishTrue+left_
 handedTrue+drug_abuseTRUE+cancerTRUE+diabetesTRUE+dizzinessTRUE+heart_diseaseT
 RUE+hypertensionTRUE+loss_of_consciousnessTRUE+seizuresTRUE+smokingTRUE+stroke
 TRUE,data = m.out,model = 'ls',bootstrap=1000)
# sink('sexFemale_60_summary.txt', append=FALSE, split=FALSE)
# summary(z.out.sexFemale)
# sink()
# z.att.sexFemale_60 = z.out.sexFemale %>% ATT(treatment = 'sexFemale', treat = 1) %>%
 get_qi(qi = 'ATT', xvalue = 'TE')
# final.df[n,"variable"] = "sexFemale_60"
# final.df[n,"ATT"] = mean(z.att.sexFemale_60)
# plot(m.out,type='hist')
# hist(z.att.sexFemale_60,main = 'sexFemale_60',xlab='ATT')
# dev.off()
# #
# #
# m.data=match.data(m.out)
```

```
# ss = nrow(match.data(m.data))
# sd_ = sd(z.att.sexFemale_60)
# se = sd_/sqrt(ss)
# final.df[n,"sample_size"] = ss
# final.df[n,"SD"] = sd_
# final.df[n,"SE"] = se
# n=n+1
# #
# View(final.df)
# rm(z.out.sexFemale)
# #
# sex_60.df = data.frame(grp = '60',att = as.vector(z.att.sexFemale_60))
# write.table(sex_60.df,'sex_60.long.csv',sep=',',col.names=T,row.names = F)
# #
# #
# ##_70
# pdf('sexFemale_70_propensity.pdf')
# m.out = matchit(sexFemale ~
 age+marital_statussingle+marital_statusunreported+marital_statuswidowed+number_of_daily_
 medications+education_bin..20+education_bin13_19+alzheimerTRUE+hispanic_latino_or_spa
 nishTrue+left_handedTrue+drug_abuseTRUE+cancerTRUE+diabetesTRUE+dizzinessTRUE+h
 eart_diseaseTRUE+hypertensionTRUE+loss_of_consciousnessTRUE+seizuresTRUE+smoking
 TRUE+strokeTRUE,data=mc.df_70, method = "nearest",discard="both")
# z.out.sexFemale = zelig(totalcorrect ~
 age+marital_statussingle+marital_statuswidowed+number_of_daily_medications+education_bi
 n..20+education_bin13_19+sexFemale+alzheimerTRUE+hispanic_latino_or_spanishTrue+left_
 handedTrue+drug_abuseTRUE+cancerTRUE+diabetesTRUE+dizzinessTRUE+heart_diseaseT
 RUE+hypertensionTRUE+seizuresTRUE+smokingTRUE+strokeTRUE,data = m.out,model =
 'ls',bootstrap=1000)
# sink('sexFemale_70_summary.txt', append=FALSE, split=FALSE)
# summary(z.out.sexFemale)
# sink()
# z.att.sexFemale_70 = z.out.sexFemale %>% ATT(treatment = 'sexFemale', treat = 1) %>%
 get_qi(qi = 'ATT', xvalue = 'TE')
# final.df[n,"variable"] = "sexFemale_70"
# final.df[n,"ATT"] = mean(z.att.sexFemale_70)
# plot(m.out,type='hist')
# hist(z.att.sexFemale_70,main = 'sexFemale_70',xlab='ATT')
# dev.off()
# #
# #
# m.data=match.data(m.out)
# ss = nrow(match.data(m.data))
# sd_ = sd(z.att.sexFemale_70)
# se = sd_/sqrt(ss)
# final.df[n,"sample_size"] = ss
```

```
# final.df[n,"SD"] = sd_
# final.df[n,"SE"] = se
# n=n+1
# #
# View(final.df)
# rm(z.out.sexFemale)
# #
# sex_70.df = data.frame(grp = '70',att = as.vector(z.att.sexFemale_70))
# write.table(sex_70.df,'sex_70.long.csv',sep=',',col.names=T,row.names = F)
# #
#
# ##_80
# pdf('sexFemale_80_propensity.pdf')
# m.out = matchit(sexFemale ~
 age+marital_statussingle+marital_statusunreported+marital_statuswidowed+number_of_daily_
 medications+education_bin..20+education_bin13_19+alzheimerTRUE+hispanic_latino_or_spa
 nishTrue+left_handedTrue+drug_abuseTRUE+cancerTRUE+diabetesTRUE+dizzinessTRUE+h
 eart_diseaseTRUE+hypertensionTRUE+loss_of_consciousnessTRUE+seizuresTRUE+smoking
 TRUE+strokeTRUE,data=mc.df_80, method = "nearest",discard="both")
# z.out.sexFemale = zelig(totalcorrect ~
 age+marital_statussingle+marital_statuswidowed+number_of_daily_medications+education_bi
 n..20+education_bin13_19+sexFemale+alzheimerTRUE+left_handedTrue+drug_abuseTRUE+c
 ancerTRUE+diabetesTRUE+dizzinessTRUE+heart_diseaseTRUE+hypertensionTRUE+smokin
 gTRUE+strokeTRUE,data = m.out,model = 'ls',bootstrap=1000)
# sink('sexFemale_80_summary.txt', append=FALSE, split=FALSE)
# summary(z.out.sexFemale)
# sink()
# z.att.sexFemale_80 = z.out.sexFemale %>% ATT(treatment = 'sexFemale', treat = 1) %>%
 get_qi(qi = 'ATT', xvalue = 'TE')
# final.df[n,"variable"] = "sexFemale_80"
# final.df[n,"ATT"] = mean(z.att.sexFemale_80)
# plot(m.out,type='hist')
# hist(z.att.sexFemale_80,main = 'sexFemale_80',xlab='ATT')
# dev.off()
#
#
# m.data=match.data(m.out)
# ss = nrow(match.data(m.data))
# sd_ = sd(z.att.sexFemale_80)
# se = sd_/sqrt(ss)
# final.df[n,"sample_size"] = ss
# final.df[n,"SD"] = sd_
# final.df[n,"SE"] = se
# n=n+1
#
# View(final.df)
```

```
# rm(z.out.sexFemale)
#
# sex_80.df = data.frame(grp = '80',att = as.vector(z.att.sexFemale_80))
# write.table(sex_80.df,'sex_80.long.csv',sep=',',col.names=T,row.names = F)
#
#
#
#
# ########half decade

# ##_25
# pdf('sexFemale_25_propensity.pdf')
# m.out = matchit(sexFemale ~
 age+marital_statussingle+marital_statusunreported+marital_statuswidowed+number_of_daily_
 medications+education_bin..20+education_bin13_19+alzheimerTRUE+hispanic_latino_or_spa
 nishTrue+left_handedTrue+drug_abuseTRUE+cancerTRUE+diabetesTRUE+dizzinessTRUE+h
 eart_diseaseTRUE+hypertensionTRUE+loss_of_consciousnessTRUE+seizuresTRUE+smoking
 TRUE+strokeTRUE,data=mc.df_25, method = "nearest",discard="both")
# z.out.sexFemale = zelig(totalcorrect ~
 age+marital_statussingle+number_of_daily_medications+education_bin..20+education_bin13_
 19+sexFemale+alzheimerTRUE+hispanic_latino_or_spanishTrue+left_handedTrue+drug_abus
 eTRUE+cancerTRUE+diabetesTRUE+dizzinessTRUE+heart_diseaseTRUE+hypertensionTRU
 E+seizuresTRUE+smokingTRUE,data = m.out,model = 'ls',bootstrap=1000)
# sink('sexFemale_25_summary.txt', append=FALSE, split=FALSE)
# summary(z.out.sexFemale)
# sink()
# z.att.sexFemale_25 = z.out.sexFemale %>% ATT(treatment = 'sexFemale', treat = 1) %>%
 get_qi(qi = 'ATT', xvalue = 'TE')
# final.df[n,"variable"] = "sexFemale_25"
# final.df[n,"ATT"] = mean(z.att.sexFemale_25)
# plot(m.out,type='hist')
# hist(z.att.sexFemale_25,main = 'sexFemale_25',xlab='ATT')
# dev.off()
# #
# #
# m.data=match.data(m.out)
# ss = nrow(match.data(m.data))
# sd_ = sd(z.att.sexFemale_25)
# se = sd_/sqrt(ss)
# final.df[n,"sample_size"] = ss
# final.df[n,"SD"] = sd_
# final.df[n,"SE"] = se
# n=n+1
# #
# View(final.df)
# rm(z.out.sexFemale)
```

```
# #
# sex_25.df = data.frame(grp = '25',att = as.vector(z.att.sexFemale_25))
# write.table(sex_25.df,'sex_25.long.csv',sep=',',col.names=T,row.names = F)
# #
# ##_35
# pdf('sexFemale_35_propensity.pdf')
# m.out = matchit(sexFemale ~
 age+marital_statussingle+marital_statusunreported+marital_statuswidowed+number_of_daily_
 medications+education_bin..20+education_bin13_19+alzheimerTRUE+hispanic_latino_or_spa
 nishTrue+left_handedTrue+drug_abuseTRUE+cancerTRUE+diabetesTRUE+dizzinessTRUE+h
 eart_diseaseTRUE+hypertensionTRUE+loss_of_consciousnessTRUE+seizuresTRUE+smoking
 TRUE+strokeTRUE,data=mc.df_35, method = "nearest",discard="both")
# z.out.sexFemale = zelig(totalcorrect ~
 age+marital_statussingle+number_of_daily_medications+education_bin..20+education_bin13_
 19+sexFemale+alzheimerTRUE+hispanic_latino_or_spanishTrue+left_handedTrue+drug_abus
 eTRUE+cancerTRUE+diabetesTRUE+dizzinessTRUE+heart_diseaseTRUE+hypertensionTRU
 E+seizuresTRUE+smokingTRUE,data = m.out,model = 'ls',bootstrap=1000)
# sink('sexFemale_35_summary.txt', append=FALSE, split=FALSE)
# summary(z.out.sexFemale)
# sink()
# z.att.sexFemale_35 = z.out.sexFemale %>% ATT(treatment = 'sexFemale', treat = 1) %>%
 get_qi(qi = 'ATT', xvalue = 'TE')
# final.df[n,"variable"] = "sexFemale_35"
# final.df[n,"ATT"] = mean(z.att.sexFemale_35)
# plot(m.out,type='hist')
# hist(z.att.sexFemale_35,main = 'sexFemale_35',xlab='ATT')
# dev.off()
# #
# #
# m.data=match.data(m.out)
# ss = nrow(match.data(m.data))
# sd_ = sd(z.att.sexFemale_35)
# se = sd_/sqrt(ss)
# final.df[n,"sample_size"] = ss
# final.df[n,"SD"] = sd_
# final.df[n,"SE"] = se
# n=n+1
# #
# View(final.df)
# rm(z.out.sexFemale)
# #
# sex_35.df = data.frame(grp = '35',att = as.vector(z.att.sexFemale_35))
# write.table(sex_35.df,'sex_35.long.csv',sep=',',col.names=T,row.names = F)

#
# ##_45
```

```
# pdf('sexFemale_45_propensity.pdf')
# m.out = matchit(sexFemale ~
 age+marital_statussingle+marital_statusunreported+marital_statuswidowed+number_of_daily_
 medications+education_bin..20+education_bin13_19+alzheimerTRUE+hispanic_latino_or_spa
 nishTrue+left_handedTrue+drug_abuseTRUE+cancerTRUE+diabetesTRUE+dizzinessTRUE+h
 eart_diseaseTRUE+hypertensionTRUE+loss_of_consciousnessTRUE+seizuresTRUE+smoking
 TRUE+strokeTRUE,data=mc.df_45, method = "nearest",discard="both")
# z.out.sexFemale = zelig(totalcorrect ~
 age+marital_statussingle+number_of_daily_medications+education_bin..20+education_bin13_
 19+sexFemale+alzheimerTRUE+hispanic_latino_or_spanishTrue+left_handedTrue+drug_abus
 eTRUE+cancerTRUE+diabetesTRUE+dizzinessTRUE+heart_diseaseTRUE+hypertensionTRU
 E+seizuresTRUE+smokingTRUE,data = m.out,model = 'ls',bootstrap=1000)
# sink('sexFemale_45_summary.txt', append=FALSE, split=FALSE)
# summary(z.out.sexFemale)
# sink()
# z.att.sexFemale_45 = z.out.sexFemale %>% ATT(treatment = 'sexFemale', treat = 1) %>%
 get_qi(qi = 'ATT', xvalue = 'TE')
# final.df[n,"variable"] = "sexFemale_45"
# final.df[n,"ATT"] = mean(z.att.sexFemale_45)
# plot(m.out,type='hist')
# hist(z.att.sexFemale_45,main = 'sexFemale_45',xlab='ATT')
# dev.off()
# #
# #
# m.data=match.data(m.out)
# ss = nrow(match.data(m.data))
# sd_ = sd(z.att.sexFemale_45)
# se = sd_/sqrt(ss)
# final.df[n,"sample_size"] = ss
# final.df[n,"SD"] = sd_
# final.df[n,"SE"] = se
# n=n+1
# #
# View(final.df)
# rm(z.out.sexFemale)
# #
# sex_45.df = data.frame(grp = '45',att = as.vector(z.att.sexFemale_45))
# write.table(sex_45.df,'sex_45.long.csv',sep=',',col.names=T,row.names = F)
# #
# #
# ##_55
# pdf('sexFemale_55_propensity.pdf')
# m.out = matchit(sexFemale ~
 age+marital_statussingle+marital_statusunreported+marital_statuswidowed+number_of_daily_
 medications+education_bin..20+education_bin13_19+alzheimerTRUE+hispanic_latino_or_spa
 nishTrue+left_handedTrue+drug_abuseTRUE+cancerTRUE+diabetesTRUE+dizzinessTRUE+h
```

```
 eart_diseaseTRUE+hypertensionTRUE+loss_of_consciousnessTRUE+seizuresTRUE+smoking
 TRUE+strokeTRUE,data=mc.df_55, method = "nearest",discard="both")
# z.out.sexFemale = zelig(totalcorrect ~
 age+marital_statussingle+marital_statuswidowed+number_of_daily_medications+education_bi
 n..20+education_bin13_19+sexFemale+alzheimerTRUE+hispanic_latino_or_spanishTrue+left_
 handedTrue+drug_abuseTRUE+cancerTRUE+diabetesTRUE+dizzinessTRUE+heart_diseaseT
 RUE+hypertensionTRUE+loss_of_consciousnessTRUE+seizuresTRUE+smokingTRUE+stroke
 TRUE,data = m.out,model = 'ls',bootstrap=1000)
# sink('sexFemale_55_summary.txt', append=FALSE, split=FALSE)
# summary(z.out.sexFemale)
# sink()
# z.att.sexFemale_55 = z.out.sexFemale %>% ATT(treatment = 'sexFemale', treat = 1) %>%
 get_qi(qi = 'ATT', xvalue = 'TE')
# final.df[n,"variable"] = "sexFemale_55"
# final.df[n,"ATT"] = mean(z.att.sexFemale_55)
# plot(m.out,type='hist')
# hist(z.att.sexFemale_55,main = 'sexFemale_55',xlab='ATT')
# dev.off()
# #
# #
# m.data=match.data(m.out)
# ss = nrow(match.data(m.data))
# sd_ = sd(z.att.sexFemale_55)
# se = sd_/sqrt(ss)
# final.df[n,"sample_size"] = ss
# final.df[n,"SD"] = sd_
# final.df[n,"SE"] = se
# n=n+1
# #
# View(final.df)
# rm(z.out.sexFemale)
# #
# sex_55.df = data.frame(grp = '55',att = as.vector(z.att.sexFemale_55))
# write.table(sex_55.df,'sex_55.long.csv',sep=',',col.names=T,row.names = F)
# #
#
##_65
# pdf('sexFemale_65_propensity.pdf')
# m.out = matchit(sexFemale ~
 age+marital_statussingle+marital_statusunreported+marital_statuswidowed+number_of_daily_
 medications+education_bin..20+education_bin13_19+alzheimerTRUE+hispanic_latino_or_spa
 nishTrue+left_handedTrue+drug_abuseTRUE+cancerTRUE+diabetesTRUE+dizzinessTRUE+h
 eart_diseaseTRUE+hypertensionTRUE+loss_of_consciousnessTRUE+seizuresTRUE+smoking
 TRUE+strokeTRUE,data=mc.df_65, method = "nearest",discard="both")
# z.out.sexFemale = zelig(totalcorrect ~
 age+marital_statussingle+marital_statuswidowed+number_of_daily_medications+education_bi
```

```
 n..20+education_bin13_19+sexFemale+alzheimerTRUE+hispanic_latino_or_spanishTrue+left_
 handedTrue+drug_abuseTRUE+cancerTRUE+diabetesTRUE+dizzinessTRUE+heart_diseaseT
 RUE+hypertensionTRUE+loss_of_consciousnessTRUE+seizuresTRUE+smokingTRUE+stroke
 TRUE,data = m.out,model = 'ls',bootstrap=1000)
# sink('sexFemale_65_summary.txt', append=FALSE, split=FALSE)
# summary(z.out.sexFemale)
# sink()
# z.att.sexFemale_65 = z.out.sexFemale %>% ATT(treatment = 'sexFemale', treat = 1) %>%
 get_qi(qi = 'ATT', xvalue = 'TE')
# final.df[n,"variable"] = "sexFemale_65"
# final.df[n,"ATT"] = mean(z.att.sexFemale_65)
# plot(m.out,type='hist')
# hist(z.att.sexFemale_65,main = 'sexFemale_65',xlab='ATT')
# dev.off()
# #
# #
# m.data=match.data(m.out)
# ss = nrow(match.data(m.data))
# sd_ = sd(z.att.sexFemale_65)
# se = sd_/sqrt(ss)
# final.df[n,"sample_size"] = ss
# final.df[n,"SD"] = sd_
# final.df[n,"SE"] = se
# n=n+1
# #
# View(final.df)
# rm(z.out.sexFemale)
# #
# sex_65.df = data.frame(grp = '65',att = as.vector(z.att.sexFemale_65))
# write.table(sex_65.df,'sex_65.long.csv',sep=',',col.names=T,row.names = F)
# #
# #
##_75
# pdf('sexFemale_75_propensity.pdf')
# m.out = matchit(sexFemale ~
 age+marital_statussingle+marital_statusunreported+marital_statuswidowed+number_of_daily_
 medications+education_bin..20+education_bin13_19+alzheimerTRUE+hispanic_latino_or_spa
 nishTrue+left_handedTrue+drug_abuseTRUE+cancerTRUE+diabetesTRUE+dizzinessTRUE+h
 eart_diseaseTRUE+hypertensionTRUE+loss_of_consciousnessTRUE+seizuresTRUE+smoking
 TRUE+strokeTRUE,data=mc.df_75, method = "nearest",discard="both")
# z.out.sexFemale = zelig(totalcorrect ~
 age+marital_statussingle+marital_statuswidowed+number_of_daily_medications+education_bi
 n..20+education_bin13_19+sexFemale+alzheimerTRUE+hispanic_latino_or_spanishTrue+left_
 handedTrue+drug_abuseTRUE+cancerTRUE+diabetesTRUE+dizzinessTRUE+heart_diseaseT
 RUE+hypertensionTRUE+smokingTRUE+strokeTRUE,data = m.out,model =
 'ls',bootstrap=1000)
```

```
# sink('sexFemale_75_summary.txt', append=FALSE, split=FALSE)
# summary(z.out.sexFemale)
# sink()
# z.att.sexFemale_75 = z.out.sexFemale %>% ATT(treatment = 'sexFemale', treat = 1) %>%
 get_qi(qi = 'ATT', xvalue = 'TE')
# final.df[n,"variable"] = "sexFemale_75"
# final.df[n,"ATT"] = mean(z.att.sexFemale_75)
# plot(m.out,type='hist')
# hist(z.att.sexFemale_75,main = 'sexFemale_75',xlab='ATT')
# dev.off()
# #
# #
# m.data=match.data(m.out)
# ss = nrow(match.data(m.data))
# sd_ = sd(z.att.sexFemale_75)
# se = sd_/sqrt(ss)
# final.df[n,"sample_size"] = ss
# final.df[n,"SD"] = sd_
# final.df[n,"SE"] = se
# n=n+1
# #
# View(final.df)
# rm(z.out.sexFemale)
# #
# sex_75.df = data.frame(grp = '75',att = as.vector(z.att.sexFemale_75))
# write.table(sex_75.df,'sex_75.long.csv',sep=',',col.names=T,row.names = F)
# #


#
 write.table(final.df,"finalATTs_sex_halfdecades.csv",sep=",",row.names=F,col.names=T,quote
 =F)
# save.image('sex_halfdecades.RData')

# Reads in FROZEN FILE USED FOR all eLife PAL Manuscript stats
dfFROZE <-
readRDS("/Volumes/huentelman/mindcrowd2/data/mindcrowd_filtered_deident_20180815.rds")

# Reads in CURRENT NON FILTERED OR FROZEN DATA
dfNEW <- readRDS("/Volumes/huentelman/mindcrowd2/data/mindcrowd_filtered.rds")

dfFROZE2 <- dfFROZE

dfNEW2 <- dfNEW

#dfNEW Already has > 36 total correct filtered out by Matt D.
```

```
dfFROZE2 <- dfFROZE2[dfFROZE2$totalcorrect <=36, ]

ggplot(dfFROZE2, aes(age,totalcorrect)) +
  geom_point(aes(color=totalcorrect), alpha = 0.01)+
  mc_theme

#ALL LM Single Variables
lm(formula = totalcorrect ~ age + sex + marital_status + left_handed +
     education + number_of_daily_medications + diabetes + seizures +
     cancer + stroke + drug_abuse + loss_of_consciousness + dizziness +
     hypertension + heart_disease + smoking + alzheimer + race + hispanic_latino_or_spanish)

# *** Full Model ONLY USE THIS ONE UPDATED 5-2-19 ***
# *** Full Model ONLY USE THIS ONE UPDATED 5-2-19 ***
dfFROZE2$race <- relevel(dfFROZE2$race, ref="White")
mylmFullALL <- lm(formula = totalcorrect ~ age + sex + marital_status + left_handed +
                  education + number_of_daily_medications + diabetes + seizures +
                  cancer + stroke + drug_abuse + loss_of_consciousness + dizziness +
                  hypertension + heart_disease + smoking + alzheimer + race +
hispanic_latino_or_spanish +
                  education*alzheimer + sex*smoking + age*alzheimer + age*diabetes +
                  alzheimer*diabetes + age*loss_of_consciousness + sex*alzheimer +
alzheimer*heart_disease
                , data = dfFROZE2)
summary(mylmFullALL)
plot(mylmFullALL)
# *** Full Model ONLY USE THIS ONE UPDATED 5-2-19 ***
# *** Full Model ONLY USE THIS ONE UPDATED 5-2-19 ***

#FOLLOW UP APOE NEW ALAYSES FOLLOWIN NEW ABOVE FULL MODEL 5-2-19
dfFROZE2$APOE <- as.factor((dfFROZE2$APOE))
dfFROZE2$APOE <- relevel(dfFROZE2$APOE, ref="2_3")
mylmFullAPOEA <- lm(formula = totalcorrect ~ age + sex + marital_status + left_handed +
                  education + number_of_daily_medications + diabetes + seizures +
                  cancer + stroke + drug_abuse + loss_of_consciousness + dizziness +
                  hypertension + heart_disease + smoking + alzheimer + hispanic_latino_or_spanish +
                  education*alzheimer + sex*smoking + age*alzheimer + age*diabetes +
                  alzheimer*diabetes + age*loss_of_consciousness + sex*alzheimer +
alzheimer*heart_disease + APOE
                , data = dfFROZE2[dfFROZE2$age<= 65 & dfFROZE2$alzheimer == TRUE,])
summary(mylmFullAPOEA)

mylmFullAPOEe4 <- lm(formula = totalcorrect ~ age + sex + marital_status + left_handed +
                   education + number_of_daily_medications + diabetes + seizures +
                   cancer + stroke + drug_abuse + loss_of_consciousness + dizziness +
```

```
                    hypertension + heart_disease + smoking + alzheimer + hispanic_latino_or_spanish
+
                    education*alzheimer + sex*smoking + age*alzheimer + age*diabetes +
                    alzheimer*diabetes + age*loss_of_consciousness + sex*alzheimer +
alzheimer*heart_disease + APOE_alleles
                  , data = dfFROZE2[dfFROZE2$age<= 65 & dfFROZE2$alzheimer == TRUE,])
summary(mylmFullAPOEe4)
#FOLLOW UP APOE NEW ALAYSES FOLLOWIN NEW ABOVE FULL MODEL 5-2-19

# MODELS AND STUFF FOR EMMEANS
dfFROZE3 <- dfFROZE2
dfFROZE3 <- dfFROZE3[dfFROZE3$age <=65,]
dfFROZE3$education <- as.factor((dfFROZE3$education))
dfFROZE3$education <- relevel(dfFROZE3$education, ref="6")
dfFROZE3$race <- relevel(dfFROZE3$race, ref="White")

ggplot(dfFROZE3, aes(age,totalcorrect)) +
  geom_point(aes(color=totalcorrect), alpha = 0.01)+
  mc_theme


# EMM for 18-65 FH x SEX
mylmFull65EMM <- lm(formula = totalcorrect ~ age + sex + marital_status + left_handed +
                    education + number_of_daily_medications + diabetes + seizures +
                    cancer + stroke + drug_abuse + loss_of_consciousness + dizziness +
                    hypertension + heart_disease + smoking + alzheimer + race +
hispanic_latino_or_spanish +
                    education*alzheimer + age*hypertension + sex*smoking + age*alzheimer +
age*diabetes +
                    alzheimer*diabetes + age*loss_of_consciousness + sex*alzheimer
                  , data = dfFROZE3)
summary(mylmFull65EMM)

emmmylmFull65FHSex <- emmeans(mylmFull65EMM, ~ sex*alzheimer)
#emmip(mylmFull65EMM, type ~ alzheimer*sex) # size | side)
summary(emmmylmFull65FHSex)
plot(emmmylmFull65FHSex, comparisons = TRUE)
pairs(emmmylmFull65FHSex)

# EMM for 18-65 FH x EDU
mylmFull65EMM <- lm(formula = totalcorrect ~ age + sex + marital_status + left_handed +
                    education + number_of_daily_medications + diabetes + seizures +
                    cancer + stroke + drug_abuse + loss_of_consciousness + dizziness +
                    hypertension + heart_disease + smoking + alzheimer + race +
hispanic_latino_or_spanish +
```

```
            education_bin*alzheimer + age*hypertension + sex*smoking + age*alzheimer +
age*diabetes +
            alzheimer*diabetes + age*loss_of_consciousness + sex*alzheimer
           , data = dfFROZE3)
summary(mylmFull65EMM)

emmmylmFull65FHSex <- emmeans(mylmFull65EMM, ~ education_bin*alzheimer)
#emmip(mylmFull65EMM, type ~ alzheimer*sex) # size | side)
summary(emmmylmFull65FHSex)
plot(emmmylmFull65FHSex, comparisons = TRUE)
pairs(emmmylmFull65FHSex)

# EMM for 18-65 FH x DIABETES
mylmFull65EMM <- lm(formula = totalcorrect ~ age + sex + marital_status + left_handed +
            education + number_of_daily_medications + diabetes + seizures +
            cancer + stroke + drug_abuse + loss_of_consciousness + dizziness +
            hypertension + heart_disease + smoking + alzheimer + race +
hispanic_latino_or_spanish +
            education*alzheimer + age*hypertension + sex*smoking + age*alzheimer +
age*diabetes +
            alzheimer*diabetes + age*loss_of_consciousness + sex*alzheimer
           , data = dfFROZE3)
summary(mylmFull65EMM)


emmmylmFull65FHDia <- emmeans(mylmFull65EMM, ~ alzheimer*diabetes)
summary(emmmylmFull65FHDia)
plot(emmmylmFull65FHDia, comparisons = TRUE)
pairs(emmmylmFull65FHDia)

#interaction = c("poly", "consec", "consec"))
# MODELS AND STUFF FOR EMMEANS

library(lm.beta)

lm.beta(mylmFullALL)

# SES ITEMS
table(dfNEW2$sex)
table(dfNEW2$took_survey)
table(dfNEW2$ses_childhood_score)
table(dfNEW2$ses_current_score)

# #1 Full Model with ALL demographic (RACE & ETHNICITY) variables ALL AGES +
SES_Child
mylmFullSESChild <- lm(formula = totalcorrect ~ age + sex + marital_status + left_handed +
```

```
                    education + number_of_daily_medications + diabetes + seizures +
                    cancer + stroke + drug_abuse + loss_of_consciousness + dizziness +
ses_childhood_score +
                    hypertension + heart_disease + smoking + alzheimer + race +
hispanic_latino_or_spanish +
                    education*alzheimer + age*hypertension + sex*smoking + age*alzheimer +
age*diabetes +
                    alzheimer*diabetes + age*loss_of_consciousness + sex*alzheimer +
alzheimer*heart_disease
                  , data = dfNEW2)
summary(mylmFullSESChild)
plot(mylmFullSESChild)

# #2 Full Model with ALL demographic (RACE & ETHNICITY) variables ALL AGES +
SES_Current
mylmFullFullSESCurrent <- lm(formula = totalcorrect ~ age + sex + marital_status +
left_handed +
                    education + number_of_daily_medications + diabetes + seizures +
                    cancer + stroke + drug_abuse + loss_of_consciousness + dizziness +
ses_current_score +
                    hypertension + heart_disease + smoking + alzheimer + race +
hispanic_latino_or_spanish +
                    education*alzheimer + age*hypertension + sex*smoking + age*alzheimer +
age*diabetes +
                    alzheimer*diabetes + age*loss_of_consciousness + sex*alzheimer +
alzheimer*heart_disease
                    , data = dfNEW2)

summary(mylmFullFullSESCurrent)
plot(mylmFullFullSESCurrent)

# #3 Reduced Model RACE & ETHNICITY ALL AGES + SES_Childhood
mylmRedSESChild <- lm(formula = totalcorrect ~ age + sex + education + alzheimer +
                  + race + hispanic_latino_or_spanish + ses_childhood_score
                 , data = dfNEW2)
summary(mylmRedSESChild)
# #4 Reduced Model with ALL demographic (RACE & ETHNICITY) vriables ALL AGES +
SES_Current
mylmRedSESCurrent <- lm(formula = totalcorrect ~ age + sex + education + alzheimer +
                    + race + hispanic_latino_or_spanish + ses_current_score
                  , data = dfNEW2)
summary(mylmRedSESCurrent)

# #6 Full Model with ALL demographic (RACE & ETHNICITY) variables ALL AGES Early
Onset AD
mylmFullEOD  <- lm(formula = totalcorrect ~ age + sex + marital_status + left_handed +
```

```
                    education + number_of_daily_medications + diabetes + seizures +
                    cancer + stroke + drug_abuse + loss_of_consciousness + dizziness +
`ad_first_degree_relative-pre55`+
                    hypertension + heart_disease + smoking + alzheimer + race +
hispanic_latino_or_spanish +
                    education*alzheimer + age*hypertension + sex*smoking + age*alzheimer +
age*diabetes +
                    alzheimer*diabetes + age*loss_of_consciousness + sex*alzheimer +
alzheimer*heart_disease
                  , data = dfNEW2)
summary(mylmFullEOD)
table(dfNEW2$`ad_first_degree_relative-pre55`)
# Evaluations of childhood, adult, amd parents education
mylmIncomeC <- lm(formula = totalcorrect ~ age + sex + marital_status + left_handed +
                   education + number_of_daily_medications + diabetes + seizures +
                   cancer + stroke + drug_abuse + loss_of_consciousness + dizziness +
ses_childhood_income_score +
                   hypertension + heart_disease + smoking + alzheimer + race +
hispanic_latino_or_spanish +
                   education*alzheimer + age*hypertension + sex*smoking + age*alzheimer +
age*diabetes +
                   alzheimer*diabetes + age*loss_of_consciousness + sex*alzheimer +
alzheimer*heart_disease
                 , data = dfNEW2)
summary(mylmIncomeC)

mylmIncomeA <- lm(formula = totalcorrect ~ age + sex + marital_status + left_handed +
                   education + number_of_daily_medications + diabetes + seizures +
                   cancer + stroke + drug_abuse + loss_of_consciousness + dizziness +
ses_current_income_score +
                   hypertension + heart_disease + smoking + alzheimer + race +
hispanic_latino_or_spanish +
                   education*alzheimer + age*hypertension + sex*smoking + age*alzheimer +
age*diabetes +
                   alzheimer*diabetes + age*loss_of_consciousness + sex*alzheimer +
alzheimer*heart_disease
                 , data = dfNEW2)
summary(mylmIncomeA)

mylmDadEdu <- lm(formula = totalcorrect ~ age + sex + marital_status + left_handed +
                  education + number_of_daily_medications + diabetes + seizures +
                  cancer + stroke + drug_abuse + loss_of_consciousness + dizziness +
`parents_highest_level_education-father` +
                  hypertension + heart_disease + smoking + alzheimer + race +
hispanic_latino_or_spanish +
```

```
                education*alzheimer + age*hypertension + sex*smoking + age*alzheimer +
age*diabetes +
                alzheimer*diabetes + age*loss_of_consciousness + sex*alzheimer +
alzheimer*heart_disease
              , data = dfNEW2)
summary(mylmDadEdu)

mylmMomEdu <- lm(formula = totalcorrect ~ age + sex + marital_status + left_handed +
                education + number_of_daily_medications + diabetes + seizures +
                cancer + stroke + drug_abuse + loss_of_consciousness + dizziness +
`parents_highest_level_education-mother` +
                hypertension + heart_disease + smoking + alzheimer + race +
hispanic_latino_or_spanish +
                education*alzheimer + age*hypertension + sex*smoking + age*alzheimer +
age*diabetes +
                alzheimer*diabetes + age*loss_of_consciousness + sex*alzheimer +
alzheimer*heart_disease
              , data = dfNEW2)
summary(mylmMomEdu)
# Evaluations of childhood, adult, amd parents education
# SES ITEMS

#ASK Matt D to get no FH and FH but not EOAD on graph,
#EOAD Years:FINAL ggplot for MC Survery
dfNEW3 <- dfNEW2
dfNEW3 = na.omit(dfNEW3[,c('ad_first_degree_relative-pre55', 'attnRT_median', 'age', 'sex',
'totalcorrect', 'diabetes', 'alzheimer', 'number_of_daily_medications' , 'left_handed', 'smoking')])
ggplot(data = dfNEW3,aes(x=age, y=totalcorrect))+
  geom_smooth(method = lm, aes(fill=`ad_first_degree_relative-pre55`,
color=`ad_first_degree_relative-pre55`), na.rm = TRUE)+
  scale_y_continuous(expand = c(0.01,0.01), breaks = scales::pretty_breaks(n = 6))+
  scale_x_continuous(expand = c(0.01,0.01), breaks = scales::pretty_breaks(n = 10))+
  scale_color_manual(values=mc_palette)+
  scale_fill_manual(values=mc_palette)+
  labs(fill = "", color = "")+
  ylab("Total Word Pairs Correct")+
  xlab("Age (Years)")+
  jst_mc_theme+
  ggsave("eLife Resub EOAD Figure.svg",last_plot(), width=7, height=7, units="in", scale = 1)
#ASK Matt D to get no FH and FH but not EOAD on graph,
#EOAD Years:FINAL ggplot for MC Survery

# MATT D'S LOLIPOP SYNTX
df_summary <- df_tmp %>%
  group_by(age, plot_group, alzheimer, diabetes, group_label) %>%
  select(age, totalcorrect, plot_group, alzheimer, diabetes, group_label) %>%
```

```
  summarise(mean_totalcorrect = mean(totalcorrect),
            sem_totalcorrect = sd(totalcorrect)/ sqrt(length(totalcorrect)))

ggplot() +
  geom_smooth(data=df_tmp,
              aes(x=age, y=totalcorrect, fill = group_label, color = group_label),
              se=TRUE, method = "lm") +
  mc_theme +
  guides(color=FALSE, fill=guide_legend(keywidth=0.1, keyheight=0.1, default.unit="inch")) +
  scale_fill_manual(values = mc_palette) +
  scale_color_manual(values = mc_palette) +
  labs(y = "Total Word Pairs Correct", x = "Age", fill = "") +
  scale_y_continuous(breaks=seq(0, 36, 2)) +
  scale_x_continuous(breaks=seq(20, max(df$age), 5)) +
  geom_errorbar(data=df_summary, aes(x=age,
                                     ymin=mean_totalcorrect-sem_totalcorrect,
                                     ymax=mean_totalcorrect+sem_totalcorrect,
                                     width=0.5), size=0.3) +
  geom_point(data=df_summary, aes(x=age, y=mean_totalcorrect, fill=group_label), size = 1,
shape = 21)
```
