# Supplementary material for: Family history of Alzheimer’s disease alters cognition and is modified by medical and genetic factors
Source: eLife. 2019 Jun 18;8:e46179. doi: 10.7554/eLife.46179 (PMC6615857; doi:10.7554/eLife.46179)
Supplement: Supplementary file 1. — Table displaying the sample size (n) for each evaluated demographic, health, and lifestyle factor. [file elife-46179-supp1.docx]

| Supplementary file 1 |  |  |  |
| --- | --- | --- | --- |
| *Sample Sizes* | | | |
| Characteristic |  |  | *n* |
| Sex |  |  |  |
|  | Women |  | 40572 |
|  | Men |  | 24381 |
|  |  |  |  |
| Education (years) |  |  |  |
|  | 6 |  | 282 |
|  | 8 |  | 181 |
|  | 10 |  | 1177 |
|  | 12 |  | 5367 |
|  | 14 |  | 19256 |
|  | 16 |  | 22942 |
|  | 20 |  | 15752 |
|  |  |  |  |
| First-Degree  Family History | FH+ |  | 14739 |
| of Alzheimer’s | FH- |  | 50011 |
| Disease (FH) |  |  |  |
|  |  |  |  |
|  |  |  |  |
| FH x Sex | FH+ Women |  | 11119 |
|  | FH- Women |  | 29332 |
|  | FH+ Men |  | 3617 |
|  | FH- Men |  | 20678 |
|  |  |  |  |
|  |  |  |  |
| Diabetes (DI) | DI+ |  | 2950 |
|  | DI- |  | 62007 |
|  |  |  |  |
|  |  |  |  |
| FH x DI | AD- DI- |  | 47970 |
|  | AD- DI+ |  | 2041 |
|  | AD+ DI- |  | 13841 |
|  | AD+ DI+ |  | 898 |
|  |  |  |  |
| APOE Genotype | ε2/ε2 |  | 2 |
|  | ε2/ε3 |  | 31 |
|  | ε2/ε4 |  | 46 |
|  | ε3/ε3 |  | 174 |
|  | ε3/ε4 |  | 382 |
|  | ε4/ε4 |  | 35 |
