# Supplementary material for: Family history of Alzheimer’s disease alters cognition and is modified by medical and genetic factors
Source: eLife. 2019 Jun 18;8:e46179. doi: 10.7554/eLife.46179 (PMC6615857; doi:10.7554/eLife.46179)
Supplement: Supplementary file 2. — Table displaying each question that a participant was asked on the MindCrowd website, whether the question was asked before or after the PAL test, the question number, and how each question was coded for analysis via R (version 3.5.1). [file elife-46179-supp2.docx]

| Supplementary file 2 | | |
| --- | --- | --- |
| *Questions each Participant was Asked and How it was Coded in R* | | |
| Regression Table | Question | Coded in R |
| Before Testing, Question 1 | Sex: | Male, Female |
| Before Testing, Question 2 | Age: | Numeric Free Response |
| Before Testing, Question 3 | Primary Language: | 1-54 (the number assigned to one of 54 major languages) |
| Before Testing, Question 4 | Education: | 6 (Six years or less)  8 (Completed eight grade)  10 (Some high school)  12 (High school diploma)  14 (Some college)  16 (Four year degree)  20 (Post graduate degree) |
| Before Testing, Question 5 | Country: | AE, AF, AG, AL, AM, AO, AR, AT, AU, BA, BB, BD, BE, BG, BH, BM, BO, BR, BS, BZ, CA, CD, CH, CL, CM, CN, CO, CR, CY, CZ, DE, DJ, DK, DO, DZ, EC, EE, EG, ES, ET, EU, FI, FJ, FR, GB, GD, GE, GG, GM, GR, GT, GU, HK, HN, HR, HU, ID, IE, IL, IM, IN, IS, IT, JE, JM, JO, JP, KE, KN, KR, KW, KZ, LB, LK, LT, LU, LV, MA, MD, ME, MF, MK, MP, MT, MU, MV, MW, MX, MY, NC, NG, NI, NL, NO, NP, NZ, OM, PA, PE, PG, PH, PK, PL, PR, PT, PY, QA, RO, RS, RU, RW, SA, SD, SE, SG, SI, SK, SN, SR, SS,SV, SY, TC, TH, TR, TT, TW, TZ, UA, UG, US, UY, VE, VI, VN, ZA, ZM, ZW |
| After Testing, Question 1 | What is your marital status? | Single, Married, Widowed, Unreported |
| After Testing, Question 2 | Are you left or right handed? | True, False |
| After Testing, Question 3 | What is your Race? | American Indian or Alaska Native, Asian, Black/African American, Native Hawaiian/Pacific Islander, White, Mixed |
| After Testing, Question 4 | Are you of Hispanic, Latino, or Spanish origin? | True, False |
| After Testing, Question 5 | How many prescription medications do you take on a daily basis? | 0, 1, 2, 3, 4 |
| After Testing, Question 6 | Please check all of the following that apply to you. |  |
| 6a | Loss of consciousness (more than 10 minutes) | True, False |
| 6b | Seizures | True, False |
| 6c | Dizzy Spells | True, False |
| 6d | High Blood Pressure | True, False |
| 6e | Smoking | True, False |
| 6f | Diabetes | True, False |
| 6g | Heart Disease | True, False |
| 6h | Cancer | True, False |
| 6i | Stroke | True, False |
| 6j | Alcohol/Drug Abuse | True, False |
| 6k | Brain Disease and/or Memory Problems | True, False |
| After Testing, Question 7 | Have you, a sibling, or one of your parents been diagnosed with Alzheimer's disease? | True, False, Not Recorded |
|  |  |  |
